# Optimization of Production Parameters for Impact Strength of 3D-Printed Carbon/Glass Fiber-Reinforced Nylon Composite in Critical ZX Printing Orientation

**DOI:** 10.3390/polym16213006

**Published:** 2024-10-26

**Authors:** Selim Hartomacioğlu

**Affiliations:** 1 Department of Mechanical Engineering, Marmara University, 34722 Istanbul, Turkey; selimh@marmara.edu.tr; Tel.: +90-545-969-90-76; 2 Department of Mechanical Engineering, Recep Tayyip Erdoğan University, 53100 Rize, Turkey

**Keywords:** additive manufacturing, polymer composite, short carbon/glass fiber, nylon

## Abstract

Additive manufacturing (AM) methods are increasingly being adopted as an alternative for mass production. In particular, Fused Deposition Modeling (FDM) technology is leading the way in this field. However, the adhesion of the layers in products produced using FDM technology is an important issue. These products are particularly vulnerable to forces acting parallel to the layers and especially to impact strength. Most products used in the industry have complex geometries and thin walls. Therefore, solid infill is often required in production, and this production must take place in the ZX orientation. This study aims to optimize the impact strength against loads acting parallel to the layers (ZX orientation) of PA6, one of the most widely used materials in the industry. This orientation is critical in terms of mechanical properties, and the mechanical characteristics are significantly lower compared to other orientations. In this study, filaments containing pure PA6 with 15% short carbon fiber and 30% glass fiber were utilized. Additionally, the printing temperature, layer thickness and heat treatment duration were used as independent variables. An L9 orthogonal array was employed for experimental design and then each experiment was repeated three times to conduct impact strength tests. Characterization, Taguchi optimization, and factor analyses were performed, followed by fracture surface characterization by SEM. As a result, the highest impact strength was achieved with pure PA6 at 8.9 kJ/m^2^, followed by PA6 GF30 at 8.1 kJ/m^2^, and the lowest impact strength was obtained with PA6 CF15 at 6.258 kJ/m^2^. Compared to the literature and manufacturer datasheets, it was concluded that the impact strength values had significantly increased and the chosen experimental factors and their levels, particularly nozzle temperature, were effective.

## 1. Introduction

Additive manufacturing (AM) methods are rapidly establishing themselves as an alternative to mass production in the industry. The need to develop these methods aligns with swiftly changing market conditions, along with continuous revision demands due to ongoing product improvements and the concept of free design thinking, all of which has driven the shift towards additive manufacturing due to lower mold costs and mold revision expenses. Among additive manufacturing methods, Fused Deposition Modeling (FDM) technology has gained significant popularity due to the cost-effectiveness of production machines and the widespread use of polymers in the industry [1]. As this technology becomes more prevalent, ensuring that industrial products meet the expected quality characteristics has become a critical research and development topic. Although many studies have been conducted on production parameters, material compositions, and design parameters, there is a notable gap in the research focused on the dynamic behavior of these products under load. In particular, there is a lack of studies on the optimization of the impact strength of the layer interface, which is the most critical area for products requiring high impact strength. Additionally, considering that existing engineering polymers like PLA, PP, ABS, and PETG may fall short in providing enough strength on their own, there has been a rapid increase in the development of composite polymers. For example, engineering materials serve as the main matrix, and additives such as carbon fiber, glass fiber, and Kevlar fiber are incorporated to enhance the quality of characteristics [1,2,3,4,5,6,7,8,9,10,11,12,13,14,15,16,17,18,19,20,21]. Despite all the advantages of the FDM method, mechanical properties in the ZX orientation are lower compared to the XY (Flat) and XZ (On-Edge) orientations. The recommended build orientations are mainly the XY and ZX orientations [1].

In this study, the scientific research conducted to date has been thoroughly examined and detailed. The selected articles were reviewed based on their direct relevance and similarities in materials and methods. The selected studies are related to the impact strength of glass fiber or carbon fiber-reinforced materials produced using FDM techniques. In this study, pure PA6, PA6/CF15, and PA6/GF30 materials have been used. Initially, relevant articles on only pure PA materials were reviewed. In this regard, the study conducted by C. Wang et al., which focuses on the impact strength of pure PA6 material, has been examined. In this study, samples were produced using pure PA filament at nozzle temperatures of 240, 245, 250, 255, and 260 °C, as well as layer thicknesses of 0.1, 0.2, and 0.3 mm. The study examined wear resistance, tensile strength, and impact strength as well [2]. In another study conducted on pure PA material, Mishra et al. also addressed the issue of impact resistance. In the study, the impact strength, bending, and tensile behaviors of polyamide samples produced through 3D printing were investigated using experimental methods and finite element analysis. Layer thicknesses of 0.1, 0.15, and 0.2 mm were used, and the samples were validated through finite element analysis after experimental production and testing procedures [3].

Studies utilize carbon, glass, and Kevlar fibers, which are available in both short and continuous forms. Continuous fibers, in particular, require specialized equipment due to the challenges in production. However, short fiber structures are relatively easier to produce today. The literature contains studies on continuous fiber-reinforced composites using 3D printing techniques, and M.A. Caminero has conducted research in this area. Continuous carbon and Kevlar fiber-reinforced nylon composites were produced using the FDM technique, and their impact strength was investigated. In the study, the reinforcement type, layer thickness, and build orientation were considered [4]. A different study was conducted by Moreno-Nunez et al., where they produced and tested polymeric composites containing carbon fibers in the XY, XZ, XY45, and XZ45 orientations using FDM techniques. The production of Onyx/aramid-based polymeric composites containing continuous carbon fiber was carried out and their impact strength was investigated. The fiber angles were set at 0°, 45°, 90°, and 135° as production parameters. The layer thickness was 0.1mm and the infill pattern used was Gyroid [5]. Studies have tested samples with continuous fiber reinforcement that have been produced in the XY and ZX printing orientations and have been subjected to testing.

The use of short fiber-reinforced composite materials is more common in 3D printing studies. A research has been conducted in this area focusing on optimizing the impact strength of short carbon fiber-reinforced PA material. In the experiments conducted for the optimization of impact strength, layer thicknesses of 0.1, 0.15, and 0.2 mm, as well as infill densities of 80%, 85%, 90%, and 95%, were considered as input variables, while the nozzle temperature was kept constant at 270 °C and the printing speed at 40mm/s. The samples were produced in the XY orientations [6]. Another study on carbon-fiber-reinforced PA6 material was conducted, where impact strength was investigated in samples produced in the XY and ZX orientations. Linear filling (100% density) and honeycomb filling (15% density) were considered as variables. The nozzle temperature was set at 230 °C, the printing speed at 35 mm/s, and the layer thickness at 0.2 mm [7]. In some studies, both carbon and glass fiber-reinforced products have been produced and subjected to testing. Authors conducted a study on this concept and produced samples using 3D printing techniques. The printing angle was considered as a variable, while other properties were kept constant and evaluated in terms of mechanical characteristics [8]. Despite the challenges associated with the 3D printing of carbon fiber-reinforced materials, many studies have been conducted in the literature due to the advantages it offers. A study was conducted in this field and performed surface analysis. The test samples were produced using PA12 material, and the variables considered included layer thickness, printing speed, number of walls, infill density, printing orientation, infill angle, nozzle temperature, and bed temperature. Notched impact test samples were produced and the surface roughness parameters were measured [9]. In the literature, there are studies on multi-layer production in fiber-reinforced materials. For example, a study was conducted in this field. The study investigated the thermal and mechanical properties of Nylon 12-CF polymeric composites produced using 3D printing methods. After production, a copper mesh layer was applied to the sample as the base material, followed by the addition of a carbon fiber layer [10]. Another study on short carbon fibers was carried out. It examined the strain ratio, mechanical properties, and damage analysis of the fracture surfaces in products produced with short carbon fiber-reinforced nylon filament using a 3D printer. Onyx filament was used in the study, and the samples were produced. The platform temperature and printing speed were considered as variables in the study [1]. Studies have also been conducted in the literature regarding the anisotropy that occurs in short-fiber composite structures based on the orientation of 3D printing. In their concepts, the effects of anisotropy and aging processes on the mechanical properties of short carbon fiber-reinforced nylon composite structures was investigated. Experimental procedures were carried out using two different infill configurations and four separate aging periods [11]. A study on impact resistance and fracture analysis was conducted in literature. In the study, glass fiber- and carbon fiber-reinforced samples were produced under fixed topology and production parameters. The nozzle temperature was considered as a variable, with PA6 material produced at a nozzle temperature of 275 °C and PA6-GF produced at 265 °C. The samples were printed in the XY orientation [12].

The physical and mechanical properties of materials vary depending on the production method. In this context, different production methods for PA/CF composite structures have been employed and tested in the literature. A study was conducted a specific study on this topic, producing and testing using the injection molding method. The injection temperature and pressure were determined as variables in the study. Additionally, pure PA-, PA-10CF-, PA-20CF- and PA-30CF-reinforced products were produced [13].

Review studies on the mechanical properties of samples produced using FDM techniques have been conducted in the literature. Studies specially focused on impact strength have been reviewed, and the research has been evaluated. In the study, a comprehensive literature review was performed on the bending and impact strength of products produced using FDM techniques. The effect of different production parameters and materials on bending and impact strength was investigated and reported. The properties of the materials were listed and evaluated according to the literature [14].

The literature review revealed that most studies focused on the XY and XZ orientations, with no direct studies specifically addressing the ZX direction, which provides anisotropic properties. Additionally, the parameter concerning the number of outer walls used in the study is an area that is lacking in the literature. Based on this, the research focused on critical orientation and specific parameters. The additive manufacturing process, particularly in the ZX orientation, which is considered the critical printing orientation, exhibits significantly lower mechanical properties compared to those produced in other orientations. For example, in the 30% carbon fiber-reinforced PA6 material provided by BASF Corp., the tensile strength value in the XY printing orientation is reported as 46.4 MPa, while the sample produced in the ZX orientation under the same conditions is reported as 12.2 MPa. Similarly, regarding impact strength, the impact strength for samples produced in the XY direction without notches is 39.6 kJ/m^2^, whereas for samples produced in the ZX direction, this value is only 3.8 kJ/m^2^. The same situation exists for PA and PA CF15 materials as well [15,16,17].

While the printing orientation can be determined based on the load for industrial products, this poses significant problems for products subjected to multi-directional forces. It is essential to enhance the mechanical properties of products in the critical ZX printing orientation through production parameters, thermal processing, and other geometric factors [1,2,3,4,5,6,7,8,9,10,11,12,13,14,15,16,17,18,19,20,21,22,23,24,25,26,27,28,29]. In this research study, impact strength testing was conducted on test samples produced using commercial filaments, where PA6 serves as the primary matrix material, reinforced with carbon fiber and glass fiber. Parallel impact forces were applied to the layers, which is the most critical situation for impact strength, and the layer adhesion performance was detailed. The production parameters, including layer thickness, nozzle temperature, and the number of outer walls, were considered as variables, while the duration of the post-processing heat treatment was also treated as an independent variable. The dependent variable was defined as the impact strength. Furthermore, the products were examined and detailed from the perspective of fracture mechanics by SEM. The results were optimized using Taguchi analysis, a statistical experimental design method, and the performance of the method was investigated through validation experiments. Subsequently, full factorial experiments were predicted and validated.

## 2. Materials and Methods

This study utilized products from the industrial filament manufacturer BASF Corp. The materials used include BASF’s Ultrafuse PA6 as the base material, Ultrafuse PA GF30 for glass fiber-reinforced PA, and Ultrafuse PA6 CF15 for carbon fiber-reinforced PA. The technical properties of the products produced in the ZX orientation are provided in Table 1 below.

This study utilized a statistical experimental design method. Initially, a factor analysis was conducted, leading to a detailed evaluation of all parameters in the production system. Based on the literature review and scientific research, effective parameters were identified. The level values for these parameters were determined and an experimental table was created. The table includes four factors, each with three levels. Under normal conditions, 3^4^ = 81 experiments would be required; however, by using the Taguchi optimization method, an L9 orthogonal array was selected, resulting in a total of nine planned experiments. To account for variations due to uncontrollable factors in the production system, each experiment was repeated three times and average values were used. Their control groups were established in the study: pure PA6, PA6 with 30% carbon fiber reinforcement, and PA6 with 15% glass fiber reinforcement. For each control group, 27 experiments and their levels are presented in Table 2, while the experimental table created using the statistical experimental design method is shown in Table 3.

**Figure 1:**
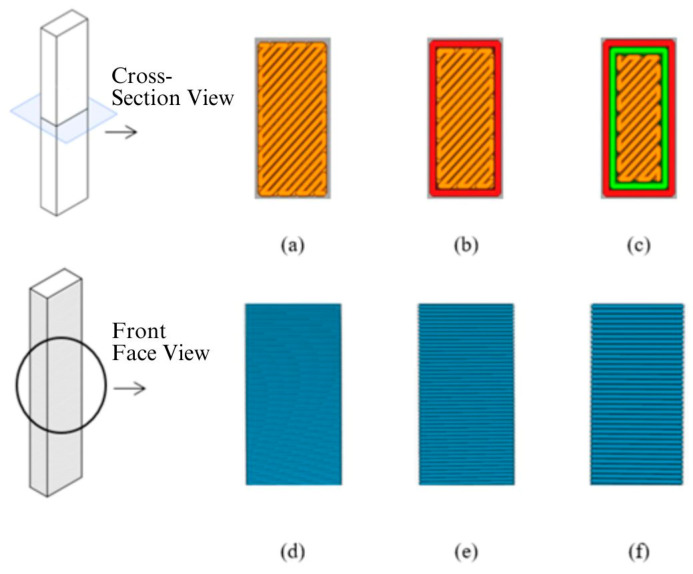
Number of outer wall number and layer thickness of samples: (**a**) wall count 0, (**b**) wall count 1, (**c**) wall count 2, (**d**) layer thickness 0.15, (**e**) layer thickness 0.30, and (**f**) layer thickness 0.45.

**Figure 2:**
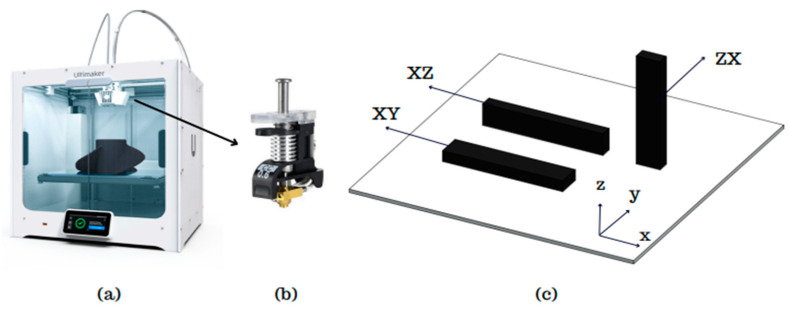
Sample production using a (**a**) 3D printer, (**b**) heat core, and (**c**) printing orientations [18].

The test samples were produced according to the ASTM 5256-10 standards [30]. The dimensions of the samples were set at 12.7 × 63.5 × 6.35 mm. To obtain parameters such as the wall thickness during the testing process, unnotched samples were prepared. The figures of prepared samples along with the production parameters of layer height and wall number are presented in Figure 1 and Figure 2.

The test samples were produced using the Ultimaker S5 printer (Ultimaker Inc., Utrecht, The Netherlands), which has a printing volume of 330 × 254 × 300 mm and features a dual extruder capable of composite production. A 0.6 CC nozzle was used for printing the composite products. Prior to production, the filament was dried for 12 h. During the printing, only the upper cover of the printer was left open, while the cover was kept closed. To ensure consistent flow before each sample’s printing, a specific period of priming was conducted.

The design of the samples was created using SolidWorks 2020 and the production planning was carried out using the Ultimaker Cura 5.3.0 (Ultimaker Inc., Utrecht, The Netherlands). In addition to the variables set during production planning, several production parameters were kept constant. The constant values of the initial layer height, infill density, infill pattern, build plate temperature, flow rate, print speed, and regular fan speed were set to 0.25 mm, 100%, lines, 70 °C, 100%, 40 mm/s, and 50%, respectively. The 3D printer, nozzle, and standard sample printing orientation used for production are shown in Figure 2.

For each experimental condition, production was repeated three times and numbered. After a general examination of the samples, they were subjected to heat treatment according to the experimental conditions. The post-processing heat treatment was applied at a temperature of 80 °C with holding times of 0, 80, and 160 min. Each sample group was then numbered and prepared for testing and other analysis.

The Izod impact test was conducted using samples prepared according to the ASTM D256-10 standards [31] on an Alarge Brand pendulum impact testing machine. The samples were tested using a 7.5 J hammer, and the energy absorbed by each sample was recorded via the device. By calculating the position of the hammer at the highest point and the energy at the last point in the impact test device, the total energy expenditure *E_T_* was found using Equation (1). The amount of total energy per unit cross-sectional area, or impact strength value, *E_C_*, was calculated using Equation (2) [12].

(1) $$E_{T}=m.g\left(h_{o}-h_{f}\right)$$

(2) $$E_{C}=\frac{E_{T}}{w.t}$$

where *E_T_* represents the total energy (J), *m* denotes mass, *g* is the standard gravitational acceleration, *h_0_* is the initial height, *hf* is the final height, *E_C_* indicates impact strength [kJ/m^2^], *w* is the sample width, and *t* is the sample thickness. Initially, in the impact testing machine, energy losses due to bearing friction and air resistance were measured and included in the calculations.

Impact strength calculations were repeated three times for each experimental condition and the average results were taken. The next step involved applying the Taguchi optimization method. In this optimization method, the first step is to calculate the signal-to-noise (S/N) ratios for each experiment. There are three different methods for calculating the S/N ratio: 1. ‘Larger is Better’, 2. ‘Smaller is Better’, and 3. ‘Target Value is Best’. In this study, since the highest value is preferable for impact strength, the S/N ratios were calculated using the ‘Larger is Better’ approach with Equation (3). During the evaluation phase, the value with the highest S/N ratio is the desired value and will be considered in the ranking [19]. Additionally, the predicted results for other experimental conditions are obtained using Equation (4) based on the values obtained.

(3) $$\frac{S}{N}=-10log\left(\frac{1}{n}\sum_{i=1}^{n}\frac{1}{Y^{2}}\right)$$

where *i = 1*,*2*,*3…n* and *Y* is the output values of response.

(4) $$\eta_{opt}=\eta_{m}+\sum ({\bar{\eta }}_{i}-\eta_{m})$$

where *η_m_* = the overall mean of signal-to-noise ratio, *f* = the number of factors, and *η_i_* = the mean of the signal-to-noise ratios at the optimal level of each factor i.

To analyze the factor effects, S/N impact graphs, S/N effect graphs, and prediction graphs for the 81 experiments and 3D surface plots to visualize the interaction effects were created. Optimal values were found for each material composition and the results were examined through validation experiments.

In the final section of the study, a damage analysis was conducted. Initially, the fracture surface of the samples was examined in their entirety using a Keyence VHX-900F (Keyence Corp., Itasca, IL, USA) optical microscope. Subsequently, a Hitachi SU-1510 Scanning Electron Microscope (SEM) (Hitachi High-Tech Corp., Ibaraki, Japan) was used for the damage analysis. Detailed images were taken post-coating, and discussion was held regarding the fracture surfaces, defects, and their causes.

## 3. Results and Discussion

In the tests, impact energies were first obtained, from which impact values were derived. Both pure and composite samples were assessed for impact energy and impact strength values. Overall, the results table shows that the highest impact energy was obtained from the pure PA6 sample, followed by the PA6 GF30 sample, while the lowest was observed in the PA CF15 sample. Literature reviews indicated that samples were generally produced in an orientation other than the critical ZX orientation. Each experiment was repeated three times under the same conditions, and the results were calculated as the arithmetic mean. The obtained impact energy values are listed in Table 4.

At this step, the impact strength values for each sample and experimental condition have been calculated using Equation (2). The results of impact strength are listed in Table 5. It was observed that the highest impact strength value was achieved in the pure PA6 sample in experiment 8, with a value of 8.990 kJ/m^2^. In the experimental condition, the parameters were A3B2C1D3, where these values corresponded to a nozzle temperature of 275 °C, a layer thickness of 0.3 mm, an outer wall count of zero, and a post-heat treatment application duration of 160 min. It is noted in the manufacturer’s product datasheet that the impact strength in the ZX orientation printing during production is 3.2 kJ/m^2^ in the notched impact test [15]. The manufacturer has set the nozzle temperature at 245 °C for the produced samples in the testing process. In this study, a higher nozzle temperature and additional heat treatment process were applied. In another study, it was observed that the impact strength in the XY printing orientation, depending on nozzle temperature, was approximately 6 kJ/m^2^ [2]. The reason for the effect of the printing orientation on mechanical properties is related to layer adhesion. In the ZX orientation, the load on the woven layers is applied parallel to the layers, resulting in lower mechanical properties. For example, a study conducted by F. Calignano et al. has shown significant differences in impact strength between the XY and ZX orientation [7].

The second-highest impact strength value in this study was obtained in the fourth experiment produced with PA6 GF30 material, measuring 8.101 kJ/m^2^. The sample was produced under the parameters of 260 °C nozzle temperature, 0.15 mm layer thickness, outer wall counts of one, and a post-heat treatment duration of 160 min, labeled as A2B1C2D3. According to the product’s datasheet, a notched Izod impact test in the ZX orientation yielded an impact strength of 2.6 kJ/m^2^. In contrast, the same sample produced in the XY orientation under identical conditions had an impact strength of 38.4 kJ/m^2^. The manufacturer’s datasheet also indicates that mechanical properties are orientation-dependent; for instance, while the tensile strength in the XY production direction is 78.3 MPa, it drops to 14.9 MPa in the ZX direction. When compared to pure PA6, these values are approximately 10% lower. However, literature studies show that the results for GF-reinforced PA materials in XY and XZ orientations are significantly higher than those for pure PA6. For example, in a study conducted by B.A. Moreno-Nunez et al., it was reported that in PA/CF materials, different production parameters and conditions achieved an impact value of 113.4 kJ/m^2^ in the XY orientation [5].

In the carbon fiber (CF)-reinforced PA6 material, the lowest impact strength value was obtained under the conditions of the seventh experiment, measuring 6.258 kJ/m^2^. This value was achieved with a nozzle temperature of 275 °C, a layer thickness of 0.15 mm, outer wall count of two, and an 80 min post-heat treatment duration. When compared to pure PA6 material, this value is approximately 30% lower, while it is about 22% lower compared to PA6 GF30 material. However, according to the datasheet provided by the manufacturer, this value was initially 2.6 kJ/m^2^, but experimental results reached 6.258 kJ/m^2^, representing an increase of approximately 2.4 times [17].

Mechanical properties indicate that if the products had been printed in the XY orientation, the expected ranking would be PA CF15 > PA6 GF30 > PA6. In a study conducted by Mohammedizadeh et al., nylon materials reinforced with carbon fiber, glass fiber, and Kevlar fiber were produced using 3D printing and their tensile strengths were examined. The study found that the highest tensile strength was exhibited by the PA/CF composite, followed by PA/GF, while the lowest value was recorded for the PA/Kevlar composite [20]. However, in the ZX orientation, which is the critical printing orientation, the ranking observed was PA6 > PA6 GF30 > PA6 CF15. The reason for this situation is explained in detail in the damage analysis section. For example, a study in evaluated these three materials in terms of the impact energy they absorbed. It was reported that the PA/GF composite stored 18.6% more energy than the PA/CF material and 210.56% more energy than the PA6 material. Thus, in this study, the ranking was also stated as PA6/GF > PA6/CF > PA6 [12]. The detailing of the printing orientation parameter is explained in a review study prepared by Solomon et al. The study indicates that the printing orientation has a significant impact on mechanical failure, with a reported 60% reduction in the ZX orientation [21]. Mechanical properties vary depending on different production methods and the parameters associated with each method. For example, in a study conducted by Wang et al., PA-CF materials were produced using injection molding techniques varying additive ratios, and combinations of PA-30CF, PA-20CF, PA-10CF, and PA were tested. The PA-30CF material achieved a tensile strength of approximately 230 MPa, while the PA-20CF material reached around 200 MPa. In contrast, the PA-10CF material had a tensile strength of about 125 MPA. The pure PA material, on the other hand, exhibited a tensile strength of approximately 50 MPa [13]. These values are significantly higher than those of the samples produced with 3D printing techniques, especially for PA/CF combinations. However, the value for the pure PA material is approximately similar when produced using 3D printing. In the literature, for example, a study reported that the tensile strength of PA/CF material produced via 3D printing reached values of 89.3 MPa [9]. In a study focused on the production of PA6 material using a different manufacturing technique, Selecting Laser Sintering (SLS), materials with 30% glass bead and 10% glass fiber content were produced. The highest tensile strength obtained was approximately 85 MPa [22]. This indicates that comprehensive research and development efforts are necessary for products produced with 3D printing technology.

**Figure 3:**
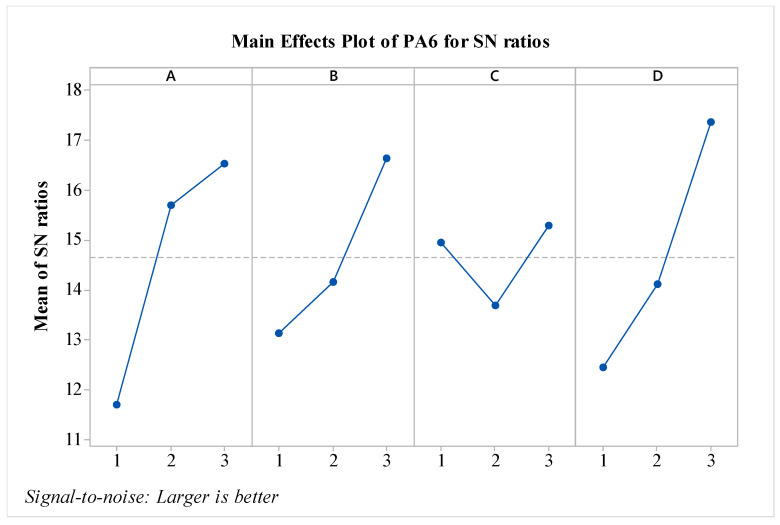
S/N ratios for PA6 material.

**Figure 4:**
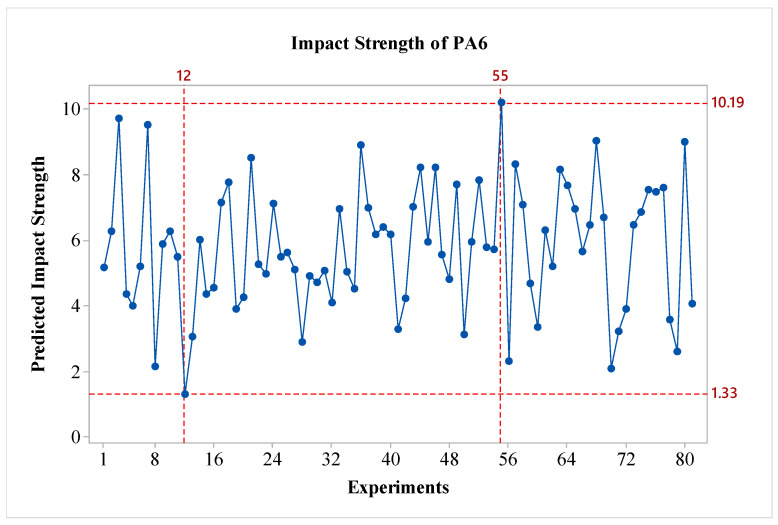
Predicted Impact Strength of PA6.

In the study, after obtaining the results, an analysis was conducted using the Taguchi method, which is one of the statistical experimental design methods widely used in engineering applications. In the literature, particularly in studies focused on impact strength, it has been stated that the Taguchi method is an effective approach [23]. Through the Taguchi analysis, the signal-to-noise (S/N) ratios for each experimental condition with different materials were calculated and graphed. The analysis was initially performed for the pure PA6 material. In the optimization of impact strength, the ‘Larger is Better’ technique was used. In terms of S/N ratios, the experiment with the highest S/N ratio is considered the most significant. In the experimental study, the S/N ratio is used as a quality parameter to evaluate the effect of input parameters on the responses [24]. The S/N ratios obtained from the experimental results for the PA6 materials are presented in Figure 3. As shown in the figure, for all factors except parameter C, the impact strength values increase as a factor level is raised. When considering the effects, it is observed that factors A and D are the most influential, while factor B is also effective, albeit to a lesser extent compared to A and D. Factor C shows a minimal effect on the results. Regarding the outer wall count, it is evident that it has a weak effect on the impact strength of PA6 materials. In this context, the literature indicates that a minimum layer thickness is desired for better bending strength, while a larger layer thickness is preferred for improved impact strength [24]. In this regard, the study is consistent with the literature on PA6 material. In terms of S/N ratios, the optimal parameters for PA6 material are identified as A3B3C2D3, which correspond to a nozzle temperature of 275 °C, a layer thickness of 0.45 mm, outer wall count of two, and a post-heat treatment duration of 160 min. For the worst-case scenario, it is observed that the A1B1C1D1 experiment corresponds to the lowest performance. Since a fractional factorial design method was used, 81 experiments were estimated using the Taguchi method’s prediction technique. The predicted results are shown in Figure 4. As seen in the figure, the difference between the highest and lowest values in the experimental design is 8.86 kJ/m^2^, indicating the effect of the factors and levels used in the experiment. The values for the best and worst cases are presented in Table 6.

**Figure 5:**
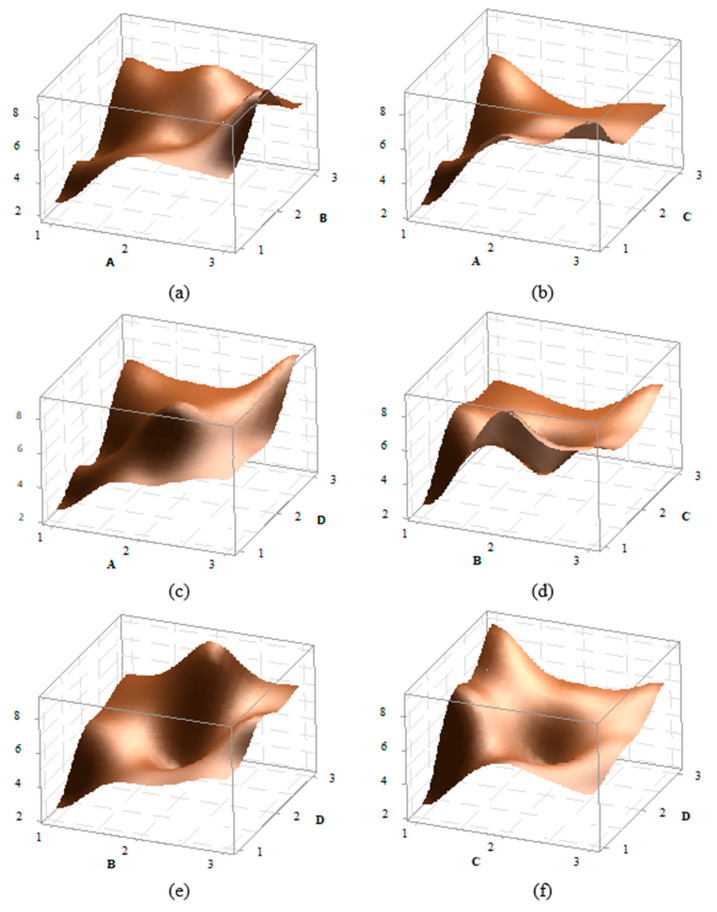
Effect graphs for PA6 material (**a**) A–B, (**b**) A–C, (**c**) A–D, (**d**) B–C, (**e**) B–D, and (**f**) C–D effect graphs.

In the statistical experimental design, the 3D graphs of factor effects are crucial for interpreting results. For the impact strength of the PA6 material, 3D effect graphs for A–B, A–C, B–C, B–D, and C–D were created and are presented in Figure 5. Upon detailed examination of the graphs, it is observed that the peak point in the A–B effect graph occurs at the A3B2 level, while the peak in the A–C graph is at the A3C1 level, in the A–D graph at A3D3, in the B–C graph at B2C1, in the B–D graph at B2D3, and in the C–D graph at C1D3. For instance, in the A–B graph, it can be seen that as a parameter increases, the impact strength also increases. Additionally, in most graphs, an increase in the D parameter also leads to an increase in the impact strength. Overall, the graphs indicate that the effects of factors and their levels are significant.

**Figure 6:**
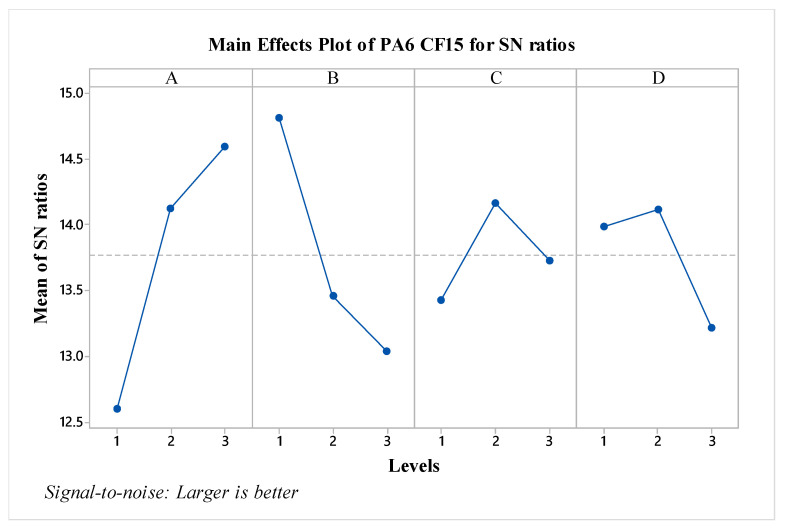
S/N ratios for PA6 CF15 material.

**Figure 7:**
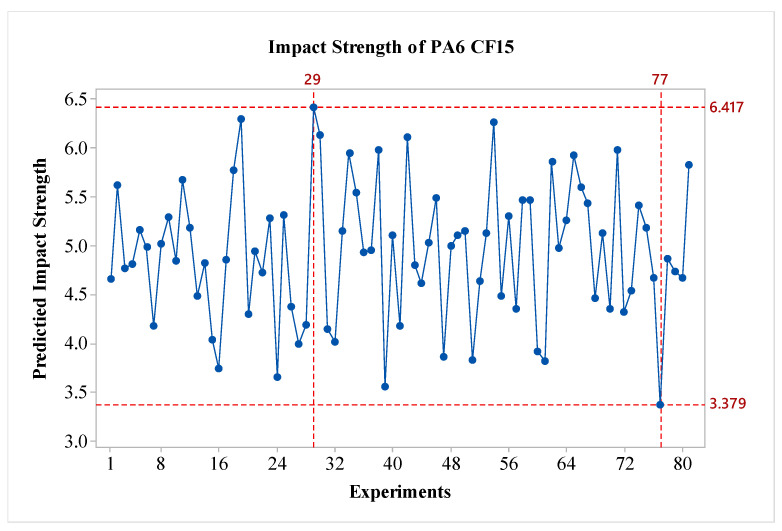
Predicted impact strength of PA6 CF15.

The S/N ratios for the PA CF15 material have been calculated and presented in Figure 6. When evaluating the factors based on S/N ratios, it is observed that factor A is the most effective, followed by factor B. In contrast to pure PA6 material, the C and D factors are seen to have minimal effect according to the graph. Nozzle temperature, also known as extrusion temperature, affects the viscosity of the material during 3D printing, thereby influencing the characteristics of the part [24]. When assessed in terms of optimal parameters, the configuration A3B1C2D2 has been identified. Particularly, compared to pure PA6, it is noted that increasing the layer thickness results in a decrease in impact strength values for PA CF15. Additionally, looking at the effect of post-heat treatment, it is observed that the impact strength level decreases after an 80 min process. The predicted results are shown in Figure 7. As seen in the figure, the difference between the highest and lowest values in the experimental design is 3.038 kJ/m2, indicating the effect of the factors and levels used in the experiment.

**Figure 8:**
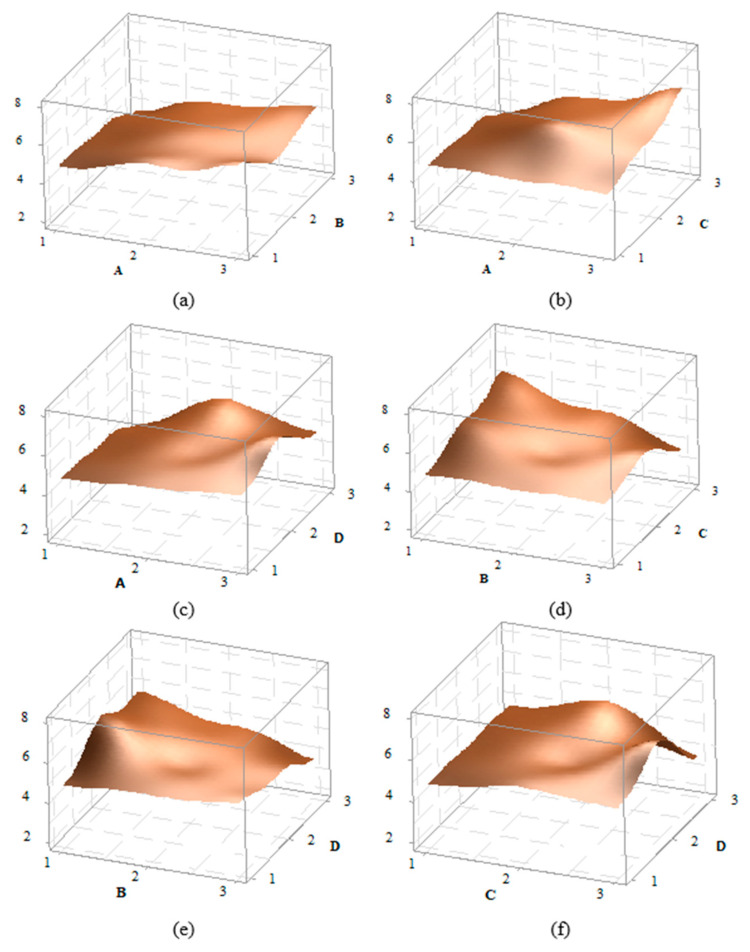
Effect graphs for PA6 CF15 material: (**a**) A–B, (**b**) A–C, (**c**) A–D, (**d**) B–C, (**e**) B–D, and (**f**) C–D effect graphs.

3D effect graphs for the impact strength value of PA6 CF15 material have been created for A–B, A–C, A–D, B–C, B–D, and C–D, and are presented in Figure 8. A detailed examination of the graphs shows that the peak point in the A–B effect graph occurs at the A3B1 level, while in the A–C graph, the peak is at the A3C3 level. In the A–D graph, the peak is at A3D2, in the B–C graph at B1C3, in the B–D graph at B1D2, and in the C–D graph at C3D2. For example, when looking at the AB graph, it is observed that as the A parameter increases, the impact strength value also increases. The D factor increases at levels 1 and 2 and decreases at level 3. Overall, the graphs indicate that the effects of the factor are significant.

**Figure 9:**
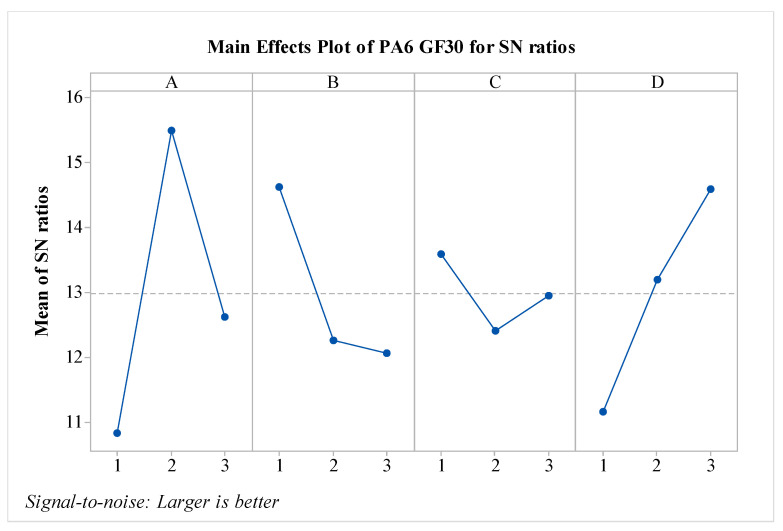
S/N ratios for PA6 GF30 material.

**Figure 10:**
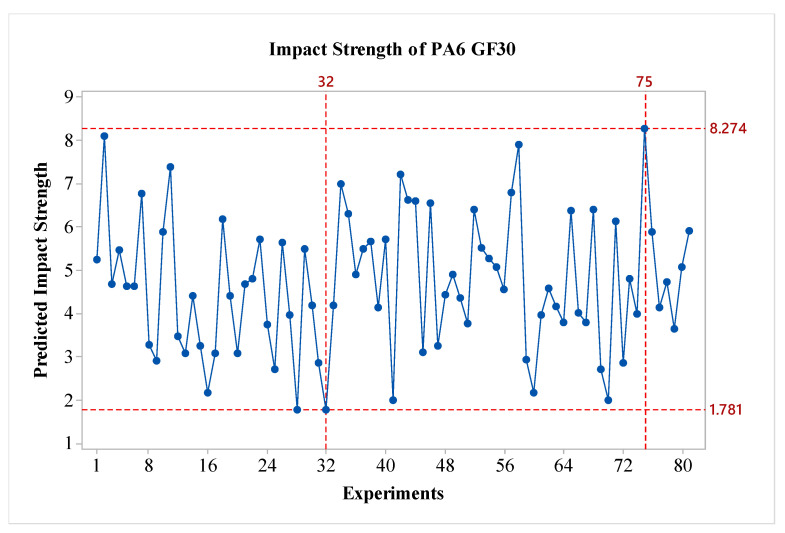
Predicted impact strength of PA6 GF30.

S/N ratios for PA6 GF30 material have been calculated and are presented in Figure 9. To determine the optimal parameters, the S/N ratios were considered. It is observed that factors A, B, and D are effective, while the effect of factor C is relatively low. The layer thickness parameter exhibited the opposite behavior compared to pure PA6 material. An experimental study on layer thickness and carbon fiber thickness was conducted by Sharma et al. The study showed that an increase in layer thickness resulted in significant decreases in tensile strength value [25]. In a similar study, it was found that in samples reinforced with carbon fiber and glass fibers, an increase in layer thickness led to a significant decrease in impact strength. Additionally, compared to the pure PA6 material, the effect of the B parameter is the opposite; that is, as the value of the B parameter increases, the impact strength decreases, like the behavior observed in PA6 GF30 material. The A parameter shows variation when compared to the PA6 CF15 material. It is anticipated that when the nozzle temperature exceeds 260 °C, the impact strength value decreases due to the embrittlement of the matrix. When evaluating the results in terms of optimal parameters, it is seen that the optimal configuration is A2B1C1D3. From the perspective of post-heat treatment, it is noted that the heat treatment duration of 160 min is effective for PA6 GF30 materials. The predicted results are shown in Figure 10. As seen in the figure, the difference between the highest and lowest values in the experimental design is 6.493 kJ/m^2^, indicating the effect of the factors and levels used in the experiment.

**Figure 11:**
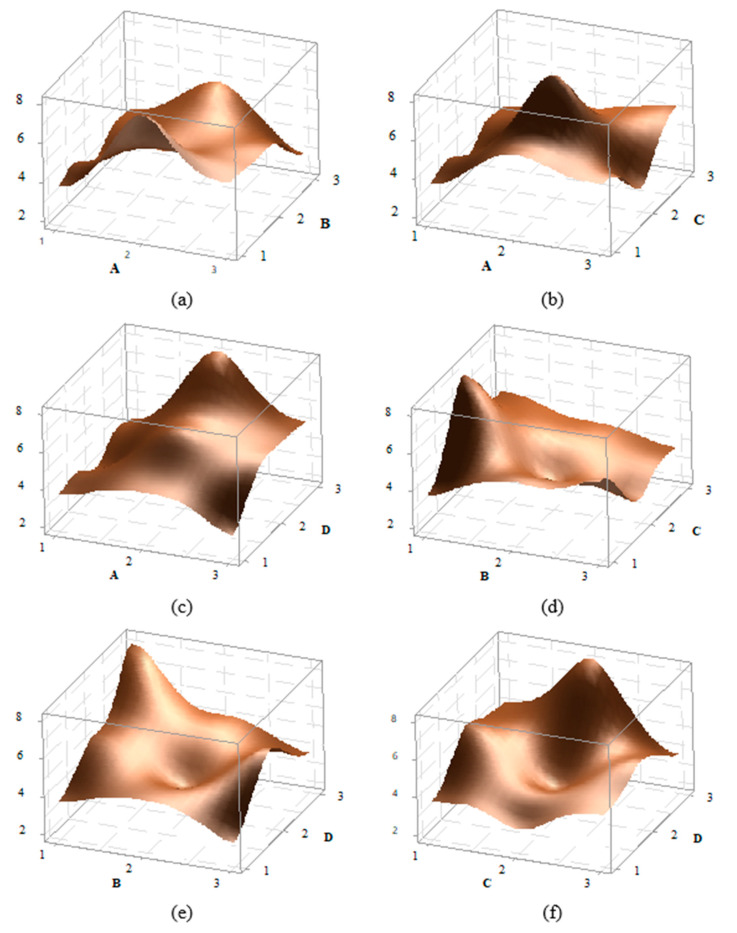
Effect graphs for PA6 GF30 material: (**a**) A–B, (**b**) A–C, (**c**) A–D, (**d**) B–C, (**e**) B–D, and (**f**) C–D effect graphs.

3D effect graphs for the impact strength value of PA6 GF30 material have been created for A–B, A–C, A–D, B–D, and C–D, and presented in Figure 11. A detailed examination of the graphs reveals that the peak point in the A–B effect graph occurs at the A2b1 level, while the peak in the A–C graph is at the A2C2 level. In the A–D graph, the peak is at A2D3, in the B–C graph at B2C2, in the B–D graph at B1D3, and in the C–D graph at C3D3. When comparing the factor effects with the S/N ratios, it is evident that they are suitable in terms of optimal values.

When examining the effects of factors on mechanical properties, it has been observed in the literature that, depending on different parameters in XY orientation weaving, the impact strength values are highest in PA/GF materials, followed by PA/CF materials. The effect of continuous carbon fiber allows the PA/CF value to reach 100 kJ/m^2^, while the effect of continuous glass fiber enables the PA/GF value to reach approximately 290 kJ/m^2^. These results indicate that the type of fiber used has a significant effect on the mechanical properties [4]. Although the continuous carbon fiber 3D printing technique provides values closest to those of PA/CF and PA/GF composites produced by the pressure injection technique, issues arise due to printing difficulties and the inability to produce desired complex surface geometries. However, with the FDM technique, it is possible to manufacture complex-shaped parts using short fiber materials, indicating a need for R&D effort in this area.

In the final step of the optimization phase of the study, the best and worst experimental conditions were identified for each material group. To determine the accuracy of the technique used, these values were additionally produced and tested, and percentage error values were calculated. The values are presented in Table 6. As seen in the table, the highest error value is 11%, while the lowest error value is 6%. The average error has been calculated as 9.51%. When considering the overall success of the prediction model, this high prediction rate is acceptable for such a complex model.

When evaluating the results overall, although the best conditions for nozzle temperature (A) are at level 3, specifically 275 °C, it is observed that for the PA6 GF30, this temperature is 260 °C. For the worst conditions, the lowest nozzle temperature is at level 1, which is 245 °C. In terms of layer thickness, it is noted that, except for pure PA6, the best value in the other material groups is provided by the lowest layer thickness of 0.15 mm. However, for pure PA6, the highest layer thickness yields the best results. When evaluating the number of outer walls (C), it is found that in optimal conditions, the wall-less structure performs best for pure PA6 and PA6/GF, whereas for PA6/CF, the structure with two walls is the best. Regarding the post-heat treatment process time (D), it has been determined that a duration of 160 min is optimal for pure PA6 and PA6 GF30 materials., while 80 min is optimal for PA6 CF156. The reason for this decrease is anticipated to be related to the thermal conductivity coefficients of the fibers. For example, while the average thermal conductivity coefficient of glass fibers is around 0.9 W/m.K, it is known that the average thermal conductivity of carbon fibers is 260 W/m.K. The literature indicates that applying heat treatment as a post-processing step leads to a significant increase in mechanical properties. In a study by Xu et al., post-processing heat treatment was applied to carbon and nylon materials in various layered products. It was reported that the optimal effect of thermal treatment on the tensile strength in a 3D-printed layered composite structure occurs at a temperature of 140–150 °C with a waiting period of 4 h, which is also optimal for interlaminar shear strength at the same temperature [26]. In this study, a post-processing heat treatment temperature of 80 °C was applied, and the results were examined. The lower temperature was chosen particularly due to reasons such as dimensional distortions and stability. In future studies, different temperatures and waiting times can be optimized based on dimensional distortions and stability characteristics.

## 4. Micro and Macro Damage Analysis

**Figure 12:**
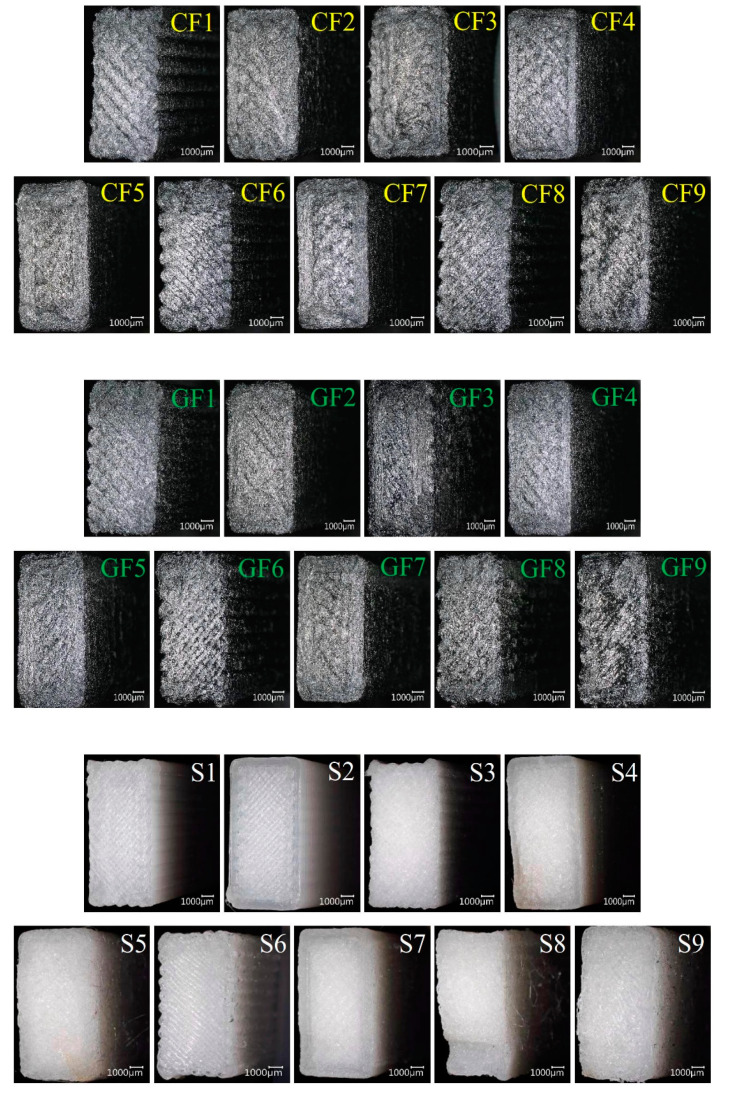
The damage images of samples produced under different parameters after impacts.

The fracture surfaces of samples are thoroughly examined using SEM for the damage analysis of composite products. In this context, various aspects such as the main matrix of the composite structure, fibers, fracture lines, fiber breakages, fiber structures, and matrix damages are analyzed in detail [27,28]. The macro damage images of the samples after impact are presented collectively in Figure 12. In the figure, view of fracture surface of 9 experiments of carbon fiber (CF)/glass fiber (GF) reinforced PA6 and pure PA6 (S) materials are given. When examining the fracture surfaces of the samples, it is observed that the pure PA6 samples exhibit a brittle fracture surface, while the fiber-reinforced samples show a more ductile and rougher surface.

**Figure 13:**
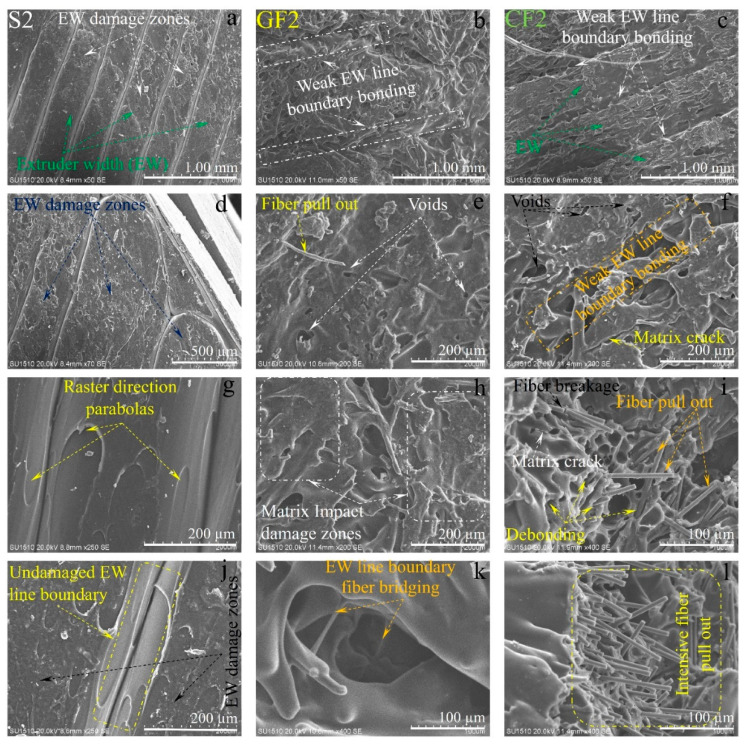
The SEM damage analysis of samples produced under second experimental conditions: (**a**,**d**,**g**,**j**) for pure PA6, (**b**,**e**,**h**,**k**) are for PA6/GF30, (**c**,**f**,**i**,**l**) for PA6 CF15 are different region and magnification).

**Figure 14:**
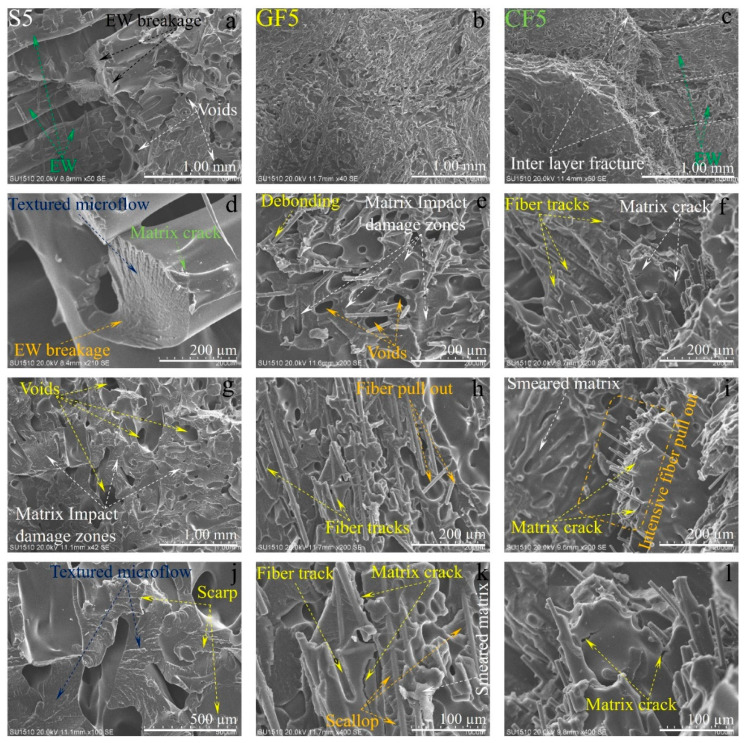
The SEM damage analysis of samples produced under fifth experimental conditions: (**a**,**d**,**g**,**j**) for pure PA6, (**b**,**e**,**h**,**k**) are for PA6/GF30, (**c**,**f**,**i**,**l**) for PA6 CF15 are different region and magnification).

**Figure 15:**
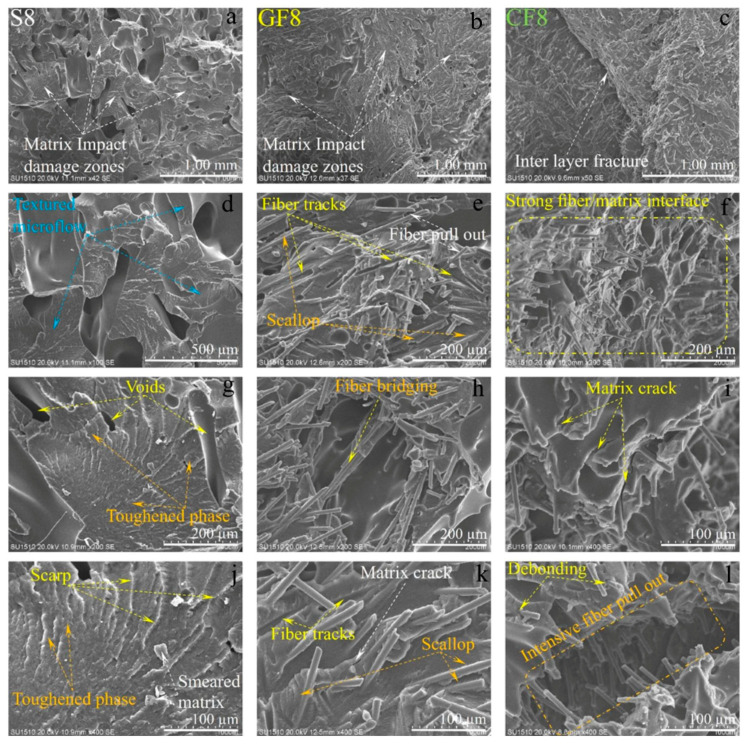
The SEM damage analysis of samples produced under eighth experimental conditions: (**a**,**d**,**g**,**j**) for pure PA6, (**b**,**e**,**h**,**k**) are for PA6/GF30, (**c**,**f**,**i**,**l**) for PA6 CF15 are different region and magnification).

The SEM images of the damaged surfaces of the samples obtained from the impact tests are presented in detail and comparatively in Figure 13, Figure 14 and Figure 15 for micro damage analyses. Similarly, in a study conducted by Pen et al., an FDM study was carried out on carbon fiber-reinforced PA6 material. The study included SEM analysis of the fracture surface of samples, demonstrating interlayer bonding, porosity, and printing bead conditions as a function of nozzle and bed temperatures [29]. Upon examining Figure 13, it can be observed that the extruder widths (EWs) are evident in pure samples, especially at low nozzle temperatures. As the nozzle temperature increases, the formation of gaps between the EWs in pure samples is noteworthy (Figure 2a). It is believed that this may be due to contraction caused by rapid cooling during the printing of the first layer. In samples reinforced with glass and carbon fibers, although there are partially weak interfacial bonds between EWs at low temperatures (Figure 13b,c), it is observed that as the nozzle temperature increases, the fibers form bridging at the boundaries between the EWs (Figure 13k). During the production phase, it is believed that the flow of the liquid matrix facilitates the flow between the EWs, helping the structure to form more homogenously (Figure 15b,c). Upon examining the fracture surfaces, it is noted that in samples of pure PA6 in the 2nd experiment, impact damages occur at the overlap surfaces of the EWs, while damage-free regions are identified at the boundary areas due to geometry (Figure 13j).

In fiber-reinforced samples (GF2, and CF2), fiber pull-out, debonding, and matrix cracks have been observed (Figure 13). Notably, intensive fiber pull-out damages have been identified in carbon fiber-reinforced samples. With the increase in temperature (Figure 14 and Figure 15), the changes in fracture surface morphology, particularly in pure PA6 samples, are striking.

On the damaged surfaces of pure PA6 samples, damage such as textured microflow, toughened phase, and scarp have been particularly observed. It is believed that the changes in damage modes and fracture surface morphology are due to heat processing. In fiber-reinforced samples, particularly those reinforced with carbon fibers, a strong fiber-matrix interfacial bond has been observed. The smaller diameter and higher surface area of carbon fiber compared to glass fibers have contributed to the formation of a stronger fiber–matrix interfacial bond. In fiber-reinforced samples, toughness mechanisms such as debonding, pull-out, matrix cracks, and fiber bridging (Figure 13, Figure 14 and Figure 15) have had a positive effect on the impact toughness of the samples.

When examining the SEM images in terms of voids, it was observed that in pure samples, large and non-homogeneous air voids formed between/in the EWs. In fiber-reinforced samples, voids appeared in certain shapes and more homogenous structures. Additionally, it was noted that in the fiber-reinforced samples, the EW line boundaries became less distinct due to fiber reinforcements, resulting in stronger boundaries compared to the pure samples. This phenomenon was attributed to fibers remaining as protrusions outside the layer during the solidification of a lower layer, which became trapped in the melt during the formation of an upper layer. This situation facilitated the formation of a good interface between the layers, thereby improving the mechanical properties of the fiber-reinforced samples.

## 5. Conclusions

In this study, optimization in terms of impact strength was performed on PA6. PA6 GF30, and PA6 CF15 polymeric composite materials produced in the ZX critical orientation, as well as on samples produced under different production parameters. The effects of the parameters were analyzed in detail. The experimental research methodology was employed in the study, and the Taguchi method, a statistical experimental design approach, was utilized. In the first stage, the production parameters and their levels, which were anticipated to influence the impact strength, were determined. Nozzle temperature, layer thickness, number of outer walls, and post-heat treatment duration were selected as the production parameters. In the next stage of the Taguchi method, the L9 orthogonal array was selected as the appropriate orthogonal array and the experimental design table was prepared. Using a 3D solid model of the test sample as the standard, the additive manufacturing process was conducted under the conditions specified in the experimental design table, and each experiment was repeated three times under the same conditions to observe variations in the production. The experimental results were obtained by calculating the arithmetic mean of the impact energy values obtained for each experiment. Subsequently, the impact strength values were calculated using the impact energy values. The S/N ratios for each experiment were calculated using the impact strength, and the effect and S/N graphs were created.

In the next phase of the study, a full factorial experimental design table was computed using the prediction formulation, and subsequently, the best and worst experimental conditions for impact strength were identified. Experiments corresponding to the best and worst condition were produced and tested, and percentage error % values were calculated.

Damage analysis is an important step in material characterization studies. For this purpose, macro and micro damage analyses were performed, with a particular focus on the effect of nozzle temperature, which is a significant parameter of impact strength. The highest impact strength value was obtained from the pure PA6 material, followed by PA6 GF30, while the lowest was from the PA6 CF15 material. It was determined that the nozzle temperature of the 3D printer was the most significant parameter and the post-processing heat treatment duration was also influential. The effect of layer thickness varied between the pure and fiber-reinforced samples: as the layer thickness increased in pure PA6, the impact strength improved, while it decreased in the others. The number of outer walls was 0 for the pure PA6 samples, whereas wall counts of one and two were effective for other materials. According to the fracture analysis results, it was observed that as nozzle temperature increased, the layer adhesion strength improved: however, due to the ZX critical direction of printing, there was no fiber connection between the two layers. This situation arises from the layer-by-layer deposition characteristics of the FDM technique, significantly leading to a reduction in mechanical properties. However, in this study, optimization of the production parameters was performed to mitigate this disadvantage, resulting in nearly three times in impact strength in samples printed in the ZX orientation. Although production planning for industrial products is typically done in the XY and XZ orientations, production must inevitably be planned in the ZX orientation for products with complex geometries. Additionally, for thin-walled parts, it is also necessary to use solid infill.

From this perspective, although this study made a significant contribution to the literature by achieving a three-times increase in mechanical properties, it has demonstrated that comprehensive studies are still needed to further improve the mechanical properties of samples produced in the ZX printing orientation. In future studies, new methods should be developed to enable fiber orientation and create connections between layers, and alternative approaches must be explored to enhance mechanical properties.

## Figures and Tables

**Table 1:** The values provided by the manufacturer of filaments [15,16,17].

Properties	Unit	PA6	PA6 CF15	PA6 GF30
Printed Part Density (dry)	Kg/m^3^	1115	1203	1356
Melting Temperature	C	195	234	209
Glass Transition Temperature	C	49	70	67
Tensile Strength	MPa	16.4	18.2	14.9
Elongation at Break	%	0.8	0.5	0.8
Young’s Modulus	MPa	2419	3532	2380
Impact Strength Izod (Unnotched)	kJ/m^2^	3.2	2.9	2.6

**Table 2:** Factors of experiments and their levels.

Factors	Unit	Level 1	Level 2	Level 3
A: Nozzle Temperature	°C	245	260	275
B: Layer Thickness	mm	0.15	0.30	0.45
C: Wall Line Count	Number	0	1	2
D: Heat Treatment Temp. Time	Min.	0	80	160

**Table 3:** L9 Statistical experimental design table.

A	B	C	D
1	1	1	1
1	2	2	2
1	3	3	3
2	1	2	3
2	2	3	1
2	3	1	2
3	1	3	2
3	2	1	3
3	3	2	1

**Table 4:** Obtained impact energy values.

Experiment	PA GF30 [J]	PA CF15 [J]	PA [J]
1	0.295	0.382	0.211
2	0.248	0.361	0.248
3	0.303	0.295	0.575
4	0.653	0.453	0.505
5	0.357	0.403	0.389
6	0.475	0.377	0.602
7	0.426	0.505	0.461
8	0.409	0.376	0.725
9	0.235	0.427	0.474

**Table 5:** Results of impact strength testing.

Experiment	PA GF30 [kJ/m^2^]	PA CF15 [kJ/m^2^]	PA [kJ/m^2^]
1	3.658	4.741	2.612
2	3.079	4.481	3.075
3	3.757	3.658	7.134
4	8.101	5.621	6.266
5	4.427	5.001	4.819
6	5.886	4.671	7.469
7	5.282	6.258	5.721
8	5.072	4.667	8.990
9	2.918	5.291	5.873

**Table 6:** The optimal and worst impact strength value and error.

Materials	Factor and Levels	Predicted Imp. Strength [kJ/m^2^]	Exp. Results of Imp. Strength [kJ/m^2^]	Error [%]
PA6_the best	A3B3C1D3	10.19	9.07	11.00
PA6_worst	A1B2C2D1	1.33	1.44	8.55
PA6 CF15_the best	A3B1C2D2	6.41	6.03	6.00
PA6 CF5_worst	A1B3C1D3	3.37	3.64	8.15
PA6 GF30_the best	A2B1C1D3	8.27	9.27	12.10
PA6_GF30_the worst	A1B3C3D1	1.78	1.63	8.30

## Data Availability

The original contributions presented in the study are included in the article. Further inquiries can be directed to the corresponding author.
